# Dietary fat intake and endometrial cancer risk: A dose response meta-analysis

**DOI:** 10.1097/MD.0000000000004121

**Published:** 2016-07-08

**Authors:** Jing Zhao, Chen Lyu, Jian Gao, Li Du, Boer Shan, Hong Zhang, Hua-Ying Wang, Ying Gao

**Affiliations:** a Key Laboratory of Nutrition and Metabolism, Institute for Nutritional Sciences, Shanghai Institutes for Biological Sciences, Chinese Academy of Sciences, University of Chinese Academy of Sciences, Shanghai, China; b Department of Applied Health Science, School of Public Health, Indiana University Bloomington, Bloomington, IN; c Department of Nutrition, Zhongshan Hospital; d Institute of Biostatistics; e Department of Gynecologic Oncology, Fudan University Shanghai Cancer Center, Department of Oncology, Shanghai Medical College, Fudan University, Shanghai, China.

**Keywords:** dose response, endometrial cancer, linoleic acid, meta-analysis, monounsaturated fatty acids, polyunsaturated fatty acids, saturated fat

## Abstract

Since body fatness is a convincing risk factor for endometrial cancer, dietary fat intake was speculated to be associated with endometrial cancer risk. However, epidemiological studies are inconclusive. We aimed to conduct a meta-analysis to assess the associations between dietary fat intake and endometrial cancer risk. We searched the PubMed, Embase, and Web of science databases updated to September 2015. In total, 7 cohort and 14 case–control studies were included. Pooled analysis of case–control studies suggested that endometrial cancer risk was significantly increased by 5% per 10% kilocalories from total fat intake (*P*=0.02) and by 17% per 10 g/1000 kcal of saturated fat intake (*P* < 0.001). Summary of 3 cohort studies showed significant inverse association between monounsaturated fatty acids and endometrial cancer risk (odds ratio = 0.84, 95% confidence interval = 0.73–0.98) with a total of 524583 participants and 3503 incident cases. No significant associations were found for polyunsaturated fatty acids and linoleic acid. In conclusion, positive associations with endometrial cancer risk were observed for total fat and saturated fat intake in the case–control studies. Results from the cohort studies suggested higher monounsaturated fatty acids intake was significantly associated with lower endometrial cancer risk.

## Abstract 2

Supplemental Digital Content is available in the text

## 1 Introduction

Endometrial cancer is one of the most common gynecologic malignancies, especially in western countries, with incidence ranking the fifth among all cancers in woman.^[1]^ Around 80% of endometrial cancers are estrogen driven.^[2]^ The most common risk factors of endometrial cancer are estrogen replacement therapy, Tamoxifen use, body fatness, diabetes mellitus, hypertension, nulliparity, infertility, early age of menarche, and late age of menopause.^[3,4]^ Among those, only body fatness, evaluated by body weight, body mass index (BMI) and waist circumference, was the only convincing risk factor of endometrial cancer according to WCRF/IARC Endometrial cancer 2013 report.^[5]^ A review commissioned by WHO showed that lower total fat intake led to lower relative body weight (by 1.6 kg), BMI (by 0.51 kg/m^2^), and waist circumference (by 0.3 cm), which might be associated with a reduction of 3% in total mortality.^[6]^ It is reasonable to speculate that excessive fat consumption may influence body fatness accumulation, and subsequently contribute to elevated risk of endometrial cancer. Studies showed that higher fat intake was linked to increased plasma estradiol, insulin secretion and IGFs levels, and inflammation markers (including hs-C-reactive protein, intercellular adhesion molecule-1, and interleukin-6), which illustrated that dietary fat intake may promote endometrial cancer development through unbalanced hormone, insulin and IGFs, and inflammation systems.^[7–11]^ These evidences suggest a possible influence of dietary fat intake on endometrial cancer risk, but to date, there is no conclusive results for the associations between fat intake and endometrial cancer risk.

Limited data suggested that total fat and saturated fat were positively associated with endometrial cancer risk.^[12]^ However, there was no enough evidence showing the effect of monounsaturated fat acids (MUFA) and polyunsaturated fat acids (PUFA) on endometrial cancer. In the current study, we aimed to evaluate dose response of total fat and saturated fat intake on endometrial cancer risk; MUFA, PUFA, and linoleic acid intake in relation to endometrial cancer risk; and effect modification of geographic region, age, BMI, and carbohydrate intake on the association.

## 2 Methods

### 2.1 Search strategy

This meta-analysis was not based on the individual participant data; ethical approval was not applicable. We systematically searched Ovid Medline, Embase, and ISI Web (up to October 22, 2014) for all the human studies on the associations between dietary fat intake and endometrial cancer risk. In addition, we screened references cited in pertinent systematic reviews. The literature was monitored by PubMed/Embase/Web of Science Alerts on predefined searched terms from October 22, 2014 to September 4, 2015.

According to WCRF Specification Manual (available at http://www.wcrf.org), the general search terms of exposure for PubMed included diet[tiab] OR diets[tiab] OR dietetic [tiab] OR dietary[tiab] OR eating[tiab] OR intake[tiab] OR nutrient^∗^[tiab] OR nutrition. In addition, the specific key words about fat included “saturated fat^∗^” OR “unsaturated fat^∗^” OR “polyunsaturated fat^∗^” OR “monounsaturated fat^∗^” OR lipid^∗^ [tiab] OR linolenic OR linoleic OR arachidonic OR eicosapentaenoic OR EPA OR docosahexaenoic OR DHA OR Docosapentaenoic OR DPA. For the outcome terms, they were consistent with those published in a previous meta-analysis^[12]^ including: (1) endometrial neoplasm[MeSH]; (2) malign^∗^ [tiab] OR cancer^∗^[tiab] OR carcinoma^∗^[tiab] OR tumor^∗^[tiab] OR tumour^∗^[tiab]; (3) endometr^∗^ [tiab] OR corpus uteri [tiab] OR uterine [tiab]; (3) #2 AND #3.

### 2.2 Study selection

**Figure 1:**
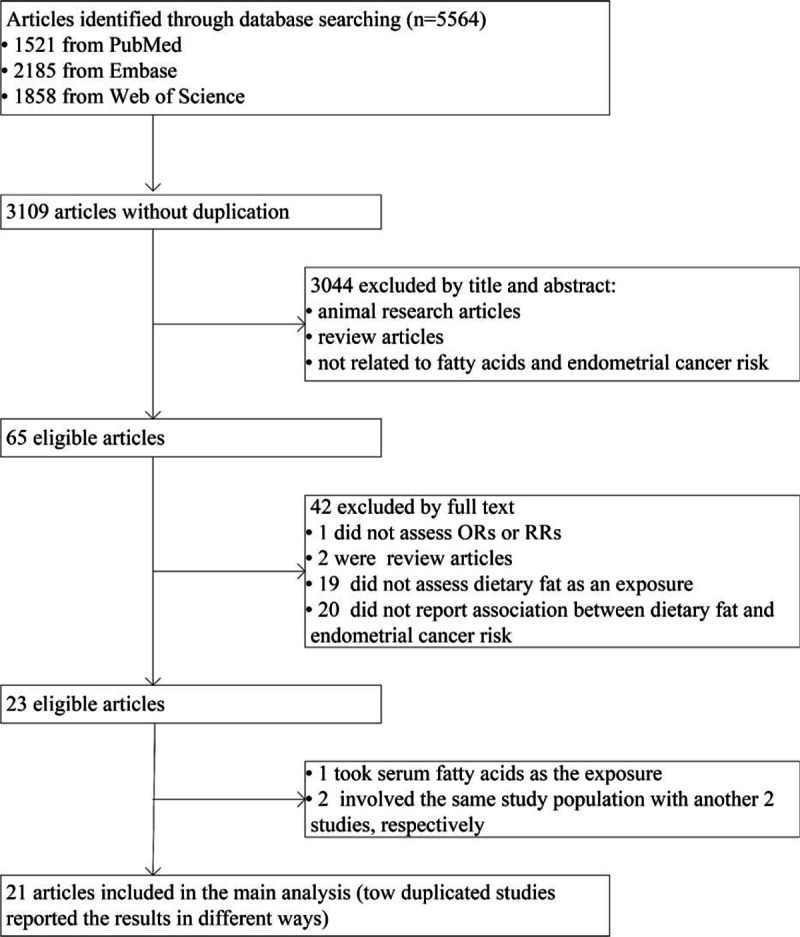
Study flow diagram.

Two investigators (JZ and CL) identified studies independently for potential inclusion. We selected studies according to the following criteria: human study: case–control study, prospective study, or clinical trial; dietary fat exposure: total fat, saturated fat, MUFA, PUFA, linoleic acid, or any other kind of fat; and outcome: endometrial cancer. After excluding duplicates of the 3 databases, there were 3109 articles left. Through further selection based on titles and abstracts, we identified 65 pertinent articles (Fig. 1). Finally, 20 articles were confirmed for the meta-analysis after reading the full text.^[13–32]^ Among the 20 articles, there were 2 pairs of duplicated study populations. For one pair of duplicate, targeting the same study population by Goodman et al.^[19–30]^ one reported odds ratios (ORs) and confidence intervals (CIs) of total fat intake and the other one reported the detailed results of saturated, monounsaturated, and polyunsaturated fat. Therefore, either of the 2 articles (but not both) was included in the specific meta-analysis. For another pair of duplicate,^[17–33]^ we chose the study with more cases (399 vs 208). Through monitoring the endometrial cancer literature after systematic screening using database alerts, we found 2 more papers published recently.^[34–35]^ In total, 21 articles were included in the meta-analysis.

### 2.3 Data extraction and assessment of study quality

Basic description data and fully adjusted estimates were carefully extracted by 2 independent investigators (JZ and CL), including sample size, age, food frequency questionnaire items, time frame, exclusion of hysterectomy, and evaluated dietary fats. The method to assess the study quality was consistent with the meta-analysis conducted in 2007.^[12]^ Briefly, we presented all the evidence from all the selected studies, and then repeated certain analyses, excluding studies that did not meet certain quality criteria. The criteria were case–control studies or cohort studies; sample size of at least 200 cases for more optimal statistical power; excluded hysterectomies from the control group; and adjusted for important confounders, such as total energy and BMI.

### 2.4 Statistical analysis

Categorical variable of fat was recruited for meta-analysis according to the WCRF criteria (more than 2 cohort or 5 case–control studies evaluated the exposure). There were enough studies to conduct meta-analysis for total fat, saturated fat, MUFA, PUFA, and linoleic acid. Inverse variance weighting method was used to calculate the summary estimate and 95%CI. The squared inverse variance for the logarithm RR/OR was considered as the appropriate weight for each study. The Q test and *I*^2^ statistics were used to examine statistical heterogeneity among studies. If *P* value was less than 0.05 for Q test or *I*^2^ index more than 25%, heterogeneity was considered statistically significant. DerSimonian and Laird random-effect model were used to pool results from all of the studies. Publication bias was evaluated with funnel plots and Egger regression asymmetry test. When *P* value was less than 0.05, the trim and fill method was used to adjust the publication bias.^[36]^ For studies without reporting of ORs or CIs, an estimate was made based on the number of cases and controls in each category of exposure.^[37]^

To explore the dose–response analysis of total fat and saturated fat with endometrial cancer risk, the method proposed by Greenland and Longnecker^[38]^ was used to compute study-specific slopes from the correlated log risk estimates across categories of dietary fat. The individual estimates were then pooled with the inverse variance weighting method to calculate the overall estimates. To estimate the dose–response trend for log RRs or log ORs across exposure categories, the generalized least squares regression model was applied.^[38]^ The mean intake of each category was calculated using the method developed by Chene and Thompson.^[39]^ For fat intake levels, we converted reported levels to nutrient density measurements expressed as either percentage of energy intake from fat (for total fat intake) or g/1000 kcal (for saturated fat intake). We also contacted the corresponding authors for sample size of exposure category or average energy intake in control groups.^[23,28]^

All statistical analyses were performed with STATA version 12.0 and R, version 3.1.2 (Package rmeta and epitools). All statistical tests were 2-sided.

## 3 Results

We identified 7 cohort studies, 11 population-based case–control studies, and 3 hospital-based case–control studies for the current meta-analysis (Fig. 1). Detailed characteristics of the studies were shown in Table 1. Among 21 studies, 14 were conducted in North America, 5 in Europe, and 2 in China. Overall, 16 studies reported total fat intake, 15 reported saturated fat, 11 reported MUFA, 9 reported PUFA, and 7 reported linoleic acid.

**Table 1:**
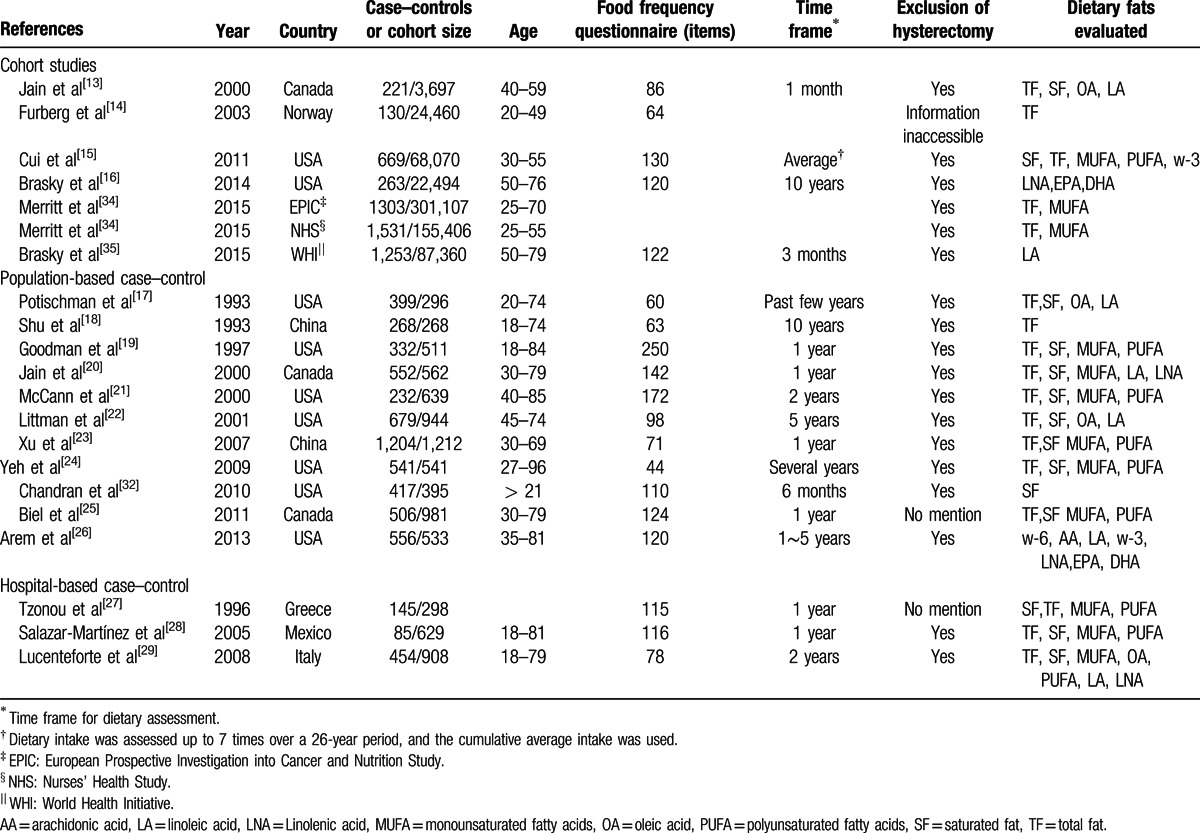
Characteristics of studies evaluating dietary fats and endometrial cancer risk.

### 3.1 Total fat

**Figure 2:**
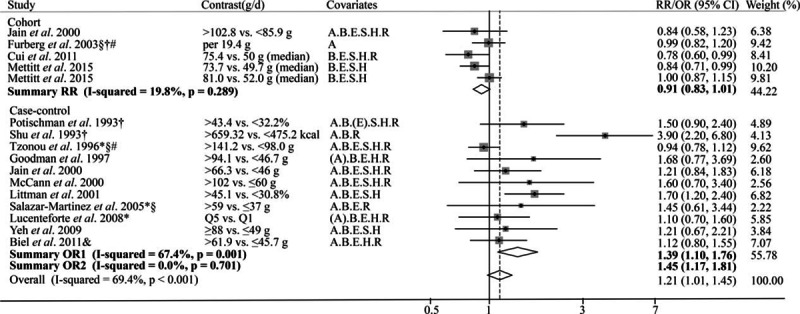
Forest plot of the summary risk estimate of endometrial cancer in the highest category of dietary total fat intake compared with those in the lowest category. Covariates: A = Age, B = BMI/weight, E = Total Energy, H = HRT/ERT use, R = Reproductive factors, S = Smoking. (A): matched on age. (E) Energy from carbohydrate calories. Weights are from random effects analysis. Summary OR1 was the summary risk estimate from all the studies. Summary OR2 was the risk estimate from studies after exclusion. Excluding studies: ^∗^Hospital-based study; §Less than 200 cases; †Exclusion of hysterectomies not clearly specified; &not adjusted for total energy intake; #not adjusted for BMI/weight.

**Figure 3:**
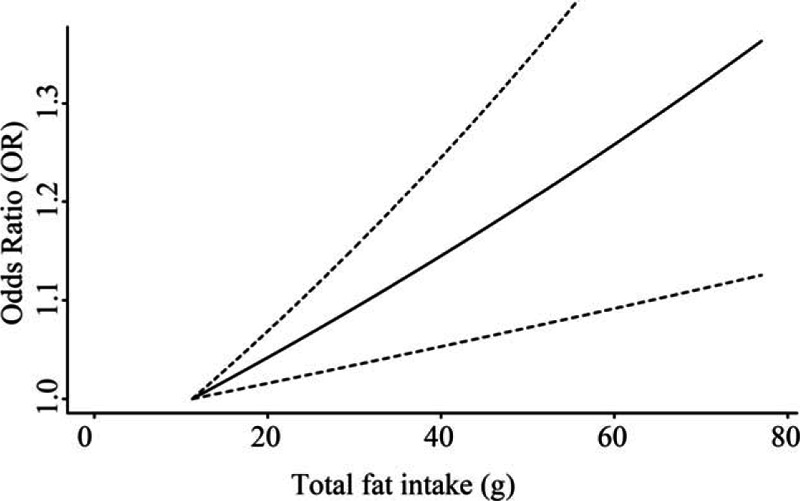
Dose–response relationship for total fat intake level and the OR of endometrial cancer for the case–control studies based on generalized least squares regression model. The smallest mean value of the lowest category interval (16% kcal from total fat intake) was used as referent baseline level (*P* for linearity = 0.015).

There were total 16 studies (5 cohorts and 11 case controls) reporting the association between total fat intake and endometrial cancer risk included in the current analysis. Based on the 5 cohort studies, marginally significant reverse association was observed between dietary total fat intake and endometrial cancer risk (pooled RR = 0.91; 95% CI = 0.83–1.01; Fig. 2) by comparing the highest category of dietary intake to the lowest category. For the 11 case–control studies, a positive association was observed (pooled OR = 1.39, 95% CI = 1.10–1.76). However, funnel plot and Egger test suggested significant publication bias (*P* = 0.01, see Figure, Supplemental Digital Content S1, which illustrates funnel plot and Egger test for 5 kinds of fat and endometrial cancer risk). When we applied the trim and fill methods to adjust for the publication bias, no significant association between total fat intake and endometrial cancer risk was observed (pooled OR = 0.96, 95% CI = 0.82–1.13) in random-effects model. After excluding studies with low quality the risk estimate was increased with OR of 1.45 (95% CI = 1.17–1.81); and Egger test showed no significant publication bias (*P* = 0.15). Meta-dose response showed that endometrial cancer risk was significantly increased by 5% when total fat intake increased 10% kilocalories from fat (*P* = 0.02; Fig. 3). Based on the limited studies, we conducted stratified analysis by geographic region, BMI, age, and carbohydrate intake. The association was stronger among individuals younger than 59, BMI less than 25, or carbohydrate intake more than 50% of total energy (see Table, Supplemental Digital Content S2, which illustrates stratified analyses of the associations between total fat or saturated fat intake and endometrial cancer risk of the case–control studies).

### 3.2 Saturated fat

**Figure 4:**
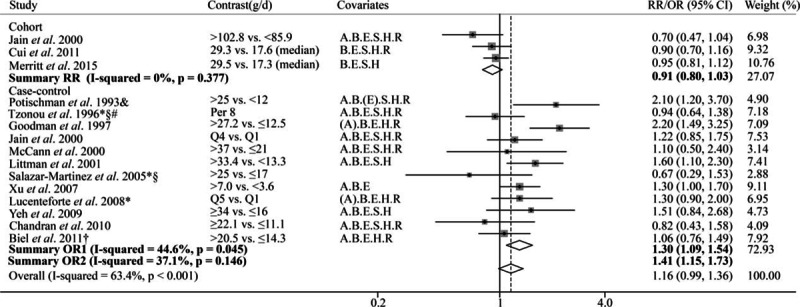
Forest plot of the summary risk estimate of endometrial cancer in the highest category of dietary saturated fat intake compared with those in the lowest category. Covariates: A = Age, B = BMI/weight, E = Total Energy, S = Smoking, H = HRT/ERT use, R = Reproductive factors. (A) Matched on age. (E) Energy from carbohydrate calories. Weights are from random effects analysis. Summary OR1 was the summary risk estimate from all the studies. Summary OR2 was the risk estimate from studies after exclusion. Excluding studies: ^∗^Hospital-based study; §Less than 200 cases; †Exclusion of hysterectomies not clearly specified; &not adjusted for total energy intake; #not adjusted for BMI/weight.

**Figure 5:**
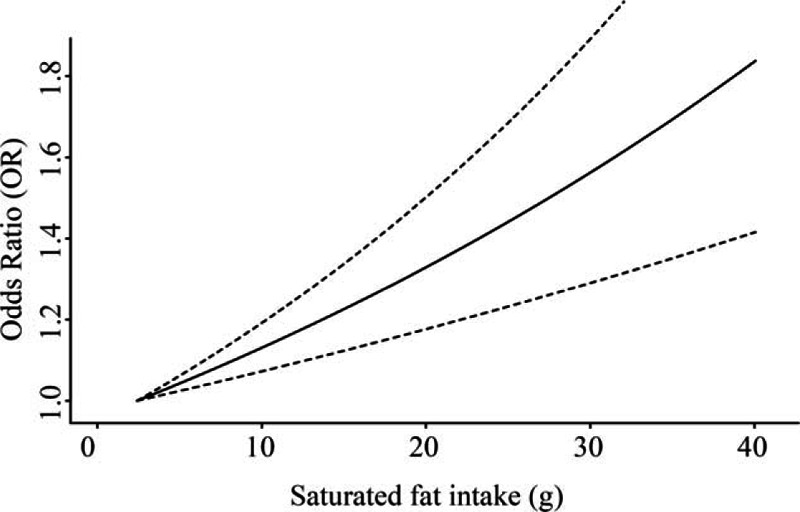
Dose–response relationship for saturated fat intake level and the OR of endometrial cancer for the case–control studies based on generalized least squares regression model. The smallest mean value of the lowest category interval (3 g per 1000 kcal) was used as referent baseline level (*P* for linearity < 0.001).

There were 15 studies that reported the association between saturated fat and endometrial cancer risk. Among them, one study (19) did not report the 95% CI, for which we adopted the estimate from a previous meta-analysis conducted in 2007 (12). Based on the meta-analysis of 3 cohort studies, when comparing those with highest intake to the lowest, marginally significant reverse association was found between dietary saturated fat intake and endometrial cancer risk (pooled RR = 0.91; 95% CI = 0.80–1.03; Fig. 4). However, for the 12 case–control studies, a positive association was observed (pooled OR = 1.30, 95% CI = 1.09–1.54); and the funnel plot and Egger test showed no significant publication bias (see Figure, Supplemental Digital Content S1, which illustrates funnel plot and Egger test for 5 kinds of fat and endometrial cancer risk). After excluding studies that did not meet the *a priori* quality criteria, the risk estimate was higher with OR of 1.41 (95% CI = 1.15–1.73); and the Egger test showed no significant publication bias (*P* = 0.70). Meta-dose response showed that endometrial cancer risk significantly increased by 17% per 10 g/1000 kcal of saturated fat intake (*P* < 0.001; Fig. 5). In the stratified analysis, the association was stronger among individuals older than 59 or with a BMI less than 25 (see Table, Supplemental Digital Content S2, which illustrates stratified analyses of the associations between total fat or saturated fat intake and endometrial cancer risk of the case–control studies).

### 3.3 Unsaturated fat

**Figure 6:**
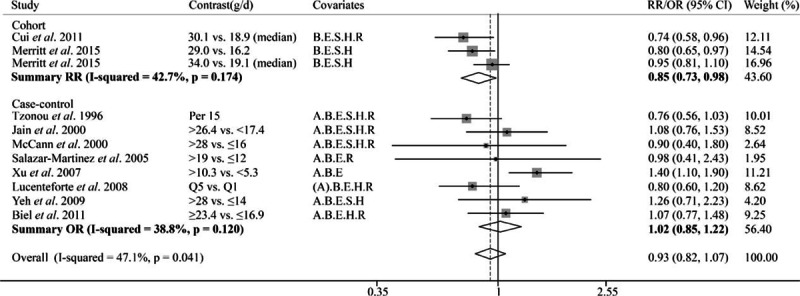
Forest plot of the summary risk estimate of endometrial cancer in the highest category of dietary monounsaturated fatty acids intake compared with those in the lowest category. The result in Goodman et al 1997 without 95% CI was excluded from the analysis. Covariates: A = Age, B = BMI/weight, E = Total Energy, S = Smoking, H = HRT/ERT use, R = Reproductive factors. (A) Matched on age. Weights are from random effects analysis.

**Figure 7:**
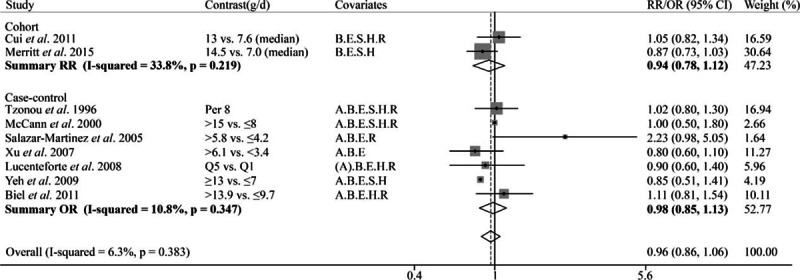
Forest plot of the summary risk estimate of endometrial cancer in the highest category of dietary polyunsaturated fatty acids intake compared with those in the lowest category. The result in Goodman et al 1997 without 95% CI was excluded from the analysis. Covariates: A = Age, B = BMI/weight, E = Total energy, S = Smoking, H = HRT/ERT use, R = Reproductive factors. (A) Matched on age. Weights are from fix effects analysis.

**Figure 8:**
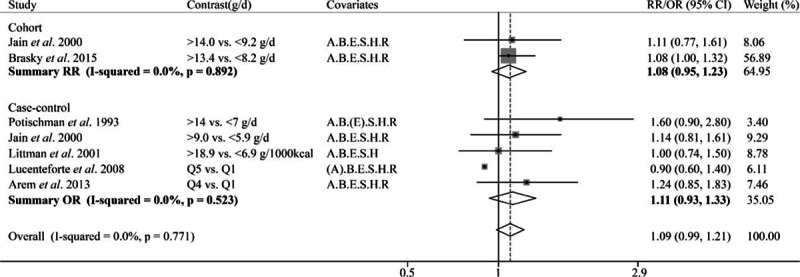
Forest plot of the summary risk estimate of endometrial cancer in the highest category of dietary linoleic acid intake compared with those in the lowest category. Covariates: A = Age, B = BMI/weight, E = Total Energy, S = Smoking, H = HRT/ERT use, R = Reproductive factors. (A) Matched on age. Weights are from fix effects analysis.

The pooled estimate of 3 cohort studies showed MUFA was negatively associated with endometrial cancer risk (OR 0.85, 95% CI = 0.73–0.98; Fig. 6). However, the meta-analysis of 8 case–control studies suggested nonsignificant association (Pooled OR = 1.02, 95% CI = 0.85–1.22). For PUFA, no association with endometrial cancer risk (Pooled OR = 0.96, 95% CI = 0.86–1.06; Fig. 7) was observed based on 2 cohort and 7 case–control studies. Two cohort and 5 case–control studies reported the association of linoleic acid and endometrial cancer risk, and the summary results suggested a marginally positive association (pooled OR = 1.09, 95% CI = 0.98–1.21; Fig. 8); and the funnel plot and Egger test did not suggest significant publication bias between unsaturated fat and cancer risk (see Figure, Supplemental Digital Content S1, which illustrates funnel plot and Egger test for 5 kinds of fat and endometrial cancer risk).

## 4 Discussion

Our meta-analysis showed that higher intake of total fat and saturated fat was significantly associated with increased endometrial cancer risk. The risk for endometrial cancer was increased by 5% when 10% kilocalories intake was increased from total fat, and by 17% when 10 g/1000 kcal calories was increased from saturated fat. A reverse association was observed between MUFA and endometrial cancer risk in cohort studies. No significant associations were observed for PUFA and linoleic acid.

Compared with the meta-analysis conducted in 2007,^[12]^ we include 9 more studies, which allowed us to explore the associations between MUFA, PUFA, linoleic acid, and endometrial cancer risk. Besides, 8 more studies were included for the analysis of total fat and saturated fat. Consistent with the study of 2007, we also observed positive associations with total and saturated fat in case–control studies, but not in cohort studies. The possible reasons include: first, there were only 5 cohort studies which might have no enough power to detect the difference. Second, bias caused by hysterectomy, which are specific for endometrial cancer in the cohort studies, should be considered. Subjects who had hysterectomy would not be at risk of endometrial cancer. However, none of the cohort studies updated the hysterectomy status during the follow up. If hysterectomy were positively associated with total and saturated fat intake, inclusion of these subjects would skew the results toward a null or even reverse association.

In the current study, we observed significant dose–response effect for total fat and saturated fat, which was not observed by a recent meta-analysis conducted by Wu.^[40]^ Our meta-analysis included one more case–control study conducted in 2007,^[23]^ which observed a positive association between saturated fat intake and endometrial cancer risk (OR = 1.3, 95% CI = 1.0–1.7). This study included 1204 cases and 1212 controls,^[23]^ which accounted for 10.4% weight in our meta-analysis. Therefore, missing this study might affect the result. In addition, we corrected the saturated fat intake with energy intake (g/1000 kcal), which was different from Wu's study using absolute intake (g/day). The total energy intake from the 15 studies involved in our meta-analysis ranged from 1227 to 2106 kcal. Even for the studies conducted in the United States, one reported the total energy intake as 1227 kcal,^[17]^ and another reported 2091 kcal.^[21]^ The large variation of total energy intake might confound the association. Certainly, the estimate variation between different questionnaires might also contribute to the discrepancy. According to Food and Agriculture Organization of the United Nations 2013 report, annual meat consumption was 120.2, 90.7, and 58.2 kg per person for United States, Italy, and China, respectively.^[41]^ Meat is the main source of saturated fat and energy. The different basic levels of meat intake might influence the association between saturated fat intake and endometrial cancer risk. Therefore, it is more rational to use total energy adjusted saturated fat.

Interestingly, we found that higher MUFA intake was significantly associated with decreased endometrial cancer risk based on the cohort studies but not case–control studies. Although animal studies suggest that MUFA could induce apoptosis and inhibit inflammation to protect against cancer,^[42]^ the results of epidemiologic studies are controversial. Many factors, including food source, food processing, and total energy intake, could influence the relationship between MUFA intake and cancer risk.^[43]^ Many studies have reported that population with higher intake of olive oil, which is rich in MUFA, had lower risk for cancer.^[44]^ Certainly, this reverse association might be confounded by highly bioactive compounds, such as tocopherols, triterpenic alcohols, plant sterols, and polyphenols in olive oil.^[45]^ If MUFA intake was primarily from red meat, which contains a great amount of saturated fat, so-called “higher MUFA intake” might increase cancer risk.^[46]^ And high-saturated fat consumers have been reported to suffer from reduced insulin sensitivity, which is a risk factor of endometrial cancer.^[47]^ Therefore, the source should be adjusted when we evaluate the effect of MUFA.

Our meta-analysis has several strengths. With enough studies, we could evaluate the dose-response effect for total fat and saturated fat; and we found both of them were associated with increased risk of endometrial cancer. Besides, this is the first time that there were enough studies to evaluate linoleic acid and endometrial cancer risk. More studies were needed for further research. There are also limitations for the present meta-analysis. First, there were no enough studies to explore the association between PUFA subtype (n-3 and n-6) and endometrial cancer risk. Second, the results are subject to the influence of measurement error linked to the nature of food frequency questionnaire. Studies with precise measurement of exposure are needed to better understand the relationship. Up to date, there is only one study applied gas chromatography/mass spectrometry (GC/MS) to determine serum linoleic acid. The result suggested that higher level of serum linoleic acid was significantly associated with decreased endometrial cancer risk, which was not observed in the meta-analysis’ results from food frequency questionnaire. Therefore, further studies were warranted to investigate the associations of fatty acids bio-markers and endometrial cancer risk. Third, small sample size in the case–control studies and few cohort studies limited the statistical power for stratified analysis. Among all the included studies, only one case–control study was conducted in Asian and 2 were conducted in Europe, which limited the extrapolation of the results. Finally, none of study analyzed different subtypes of endometrial cancer. In fact, type I endometrial cancer was estrogen dependent, and associated with endometrial hyperplasia; but type II endometrial cancer was estrogen independent and associated with endometrial atrophy.^[48]^ These differences might affect the association estimates between dietary fat intake and cancer risk.

In summary, this meta-analysis suggested that high intake of total fat and saturated fat was associated with increased endometrial cancer risk. In addition, dietary MUFA was associated with decreased risk of endometrial cancer among cohort studies. Future prospective studies with precise intake measurement are needed to evaluate the effect of different type of fat on endometrial cancer risk of type I and type II tumors.

## Acknowledgments

We thank Liangliang Kong from Shanghai Institutes for Biological Sciences for his help in search strategy consultation. We are also grateful for help from Associate Prof. Peizhan Chen at the Institute for Nutritional Sciences, Chinese Academy of Sciences, for statistics methods consultation.

## Supplementary Material

Supplemental Digital Content
