# Supplementary material for: Dietary fat intake and endometrial cancer risk: A dose response meta-analysis
Source: Medicine (Baltimore). 2016 Jul 8;95(27):e4121. doi: 10.1097/MD.0000000000004121 (PMC5058849; doi:10.1097/MD.0000000000004121)
Supplement: Supplemental Digital Content [file medi-95-e4121-s001.docx]

**Supplemental Digital Content 1.** Figure that illustrates funnel plots and Egger’s test of meta-analysis of five kinds of fat and endometrial cancer risk. (A) Total fat. (B) Saturated fat. (C) Monounsaturated fatty acids. (D) Polyunsaturated fatty acids. (E) Linoleic acid. tif


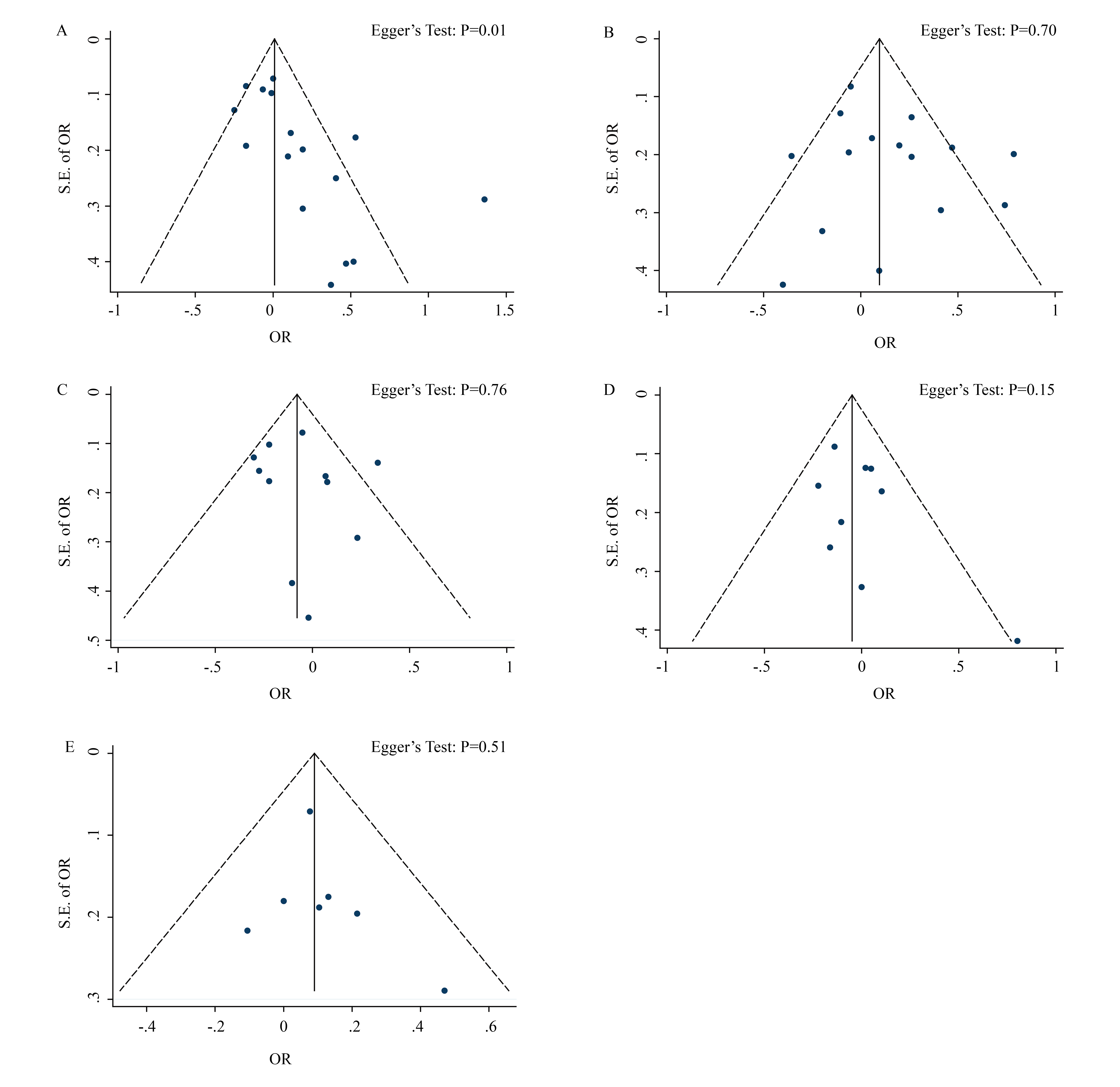


Supplemental Digital Content 2. Stratified analyses of the associations between total fat or saturated fat intake and endometrial cancer risk of the case-control studies (highest vs lowest category)

|  | **Total fat intake** | | | | **Saturated fat intake** | | | |
| --- | --- | --- | --- | --- | --- | --- | --- | --- |
| Stratification group | No. of study | *I*^2^ | *P* | OR (95% CI) | No. of study | *I*^2^ | *P* | OR (95% CI) |
| **Geographic region** |  |  |  |  |  |  |  |  |
| North America | 8 | 0.0% | 0.777 | 1.36(1.15-1.61) | 9 | 52.5% | 0.032 | 1.35(1.06-1.71) |
| Europe | 2 | 0.0% | 0.482 | 0.96(0.81-1.13) | 2 | 24.0% | 0.251 | 1.10(0.80- 1.51) |
| Asia | 1 |  |  | 3.90(2.22-6.86) | 1 |  |  | 1.30(1.00-1.69) |
| **Age** |  |  |  |  |  |  |  |  |
| <59 | 5 | 71.5% | 0.007 | 1.71(1.07-2.73) | 5 | 38.6% | 0.164 | 1.23(0.94-1.61) |
| ≥59 | 5 | 0.0% | 0.497 | 1.35(1.11-1.65) | 6 | 43.1% | 0.118 | 1.44(1.13-1.82) |
| **BMI** |  |  |  |  |  |  |  |  |
| <25 | 3 | 68.5% | 0.042 | 2.23(1.27-3.91) | 3 | 58.5% | 0.090 | 1.62(1.19-2.19) |
| ≥25 | 5 | 0.0% | 0.910 | 1.18(0.94-1.47) | 6 | 0.0% | 0.549 | 1.11(0.90-1.37) |
| **Carbohydrate Intake** |  |  |  |  |  |  |  |  |
| <50% | 5 | 65.8% | 0.102 | 1.30(0.95-1.79) | 5 | 42.8% | 0.136 | 1.38(1.03-1.86) |
| ≥50% | 6 | 69.5% | 0.033 | 1.50(1.03-2.17) | 6 | 54.4% | 0.052 | 1.29(1.03-1.63) |
